# Prevalence of Hypertension in Akwa Ibom State, South-South Nigeria: Rural versus Urban Communities Study

**DOI:** 10.1155/2015/975819

**Published:** 2015-06-10

**Authors:** Effiong Ekong Akpan, Udeme E. Ekrikpo, Aniema I. A. Udo, Bassey Edet Bassey

**Affiliations:** Department of Internal Medicine, University of Uyo Teaching Hospital, Uyo 520271, Nigeria

## Abstract

Recent studies have shown an increasing trend in the prevalence of hypertension in rural communities compared to that of the urban communities. This study was therefore carried out to determine the prevalence of hypertension and its predictors (if any) in both urban and rural communities of Akwa Ibom State of Nigeria.* Subjects and Method*. This was a cross-sectional study of urban and rural communities of Akwa Ibom State for the prevalence of hypertension and its predictors. Two urban cities and two rural communities were randomly selected from the three senatorial districts of the state. Hypertension was defined based on the Seventh Report of the Joint National Committee on the Prevention, Detection, Evaluation, and Treatment of Hypertension.* Results*. Nine hundred and seventy-eight (978) participants were recruited from rural areas and five hundred and ninety (590) from urban centers. The rural populace had higher systolic, diastolic, and mean arterial blood pressure than the urban populace (*P* < 0.001, < 0.002, < 0.001, resp.). The prevalence of hypertension was significantly higher in the rural populace than in the urban populace [44.3% (95% CI 41.1–47.4%) versus 28.6% (95% CI 24.9–32.3%)]. Age, BMI, and proteinuria were independent predictors of hypertension occurrence.* Conclusion*. There is an epidemiologic change in the prevalence of hypertension in the rural communities of Nigeria.

## 1. Introduction

Hypertension is a common and major public health problem associated with a high level cardiovascular morbidity and mortality worldwide [1]. It remains the major risk factor for heart failure, stroke, coronary artery disease, and chronic renal failure in Nigeria [2]. Hypertension which was initially considered rare in sub-Saharan Africa is now a major noncommunicable disease threatening sub-Saharan Africa. Previous studies in sub-Saharan Africa had shown a higher prevalence of hypertension in urban centers than in rural communities [3, 4], but recent studies show a growing trend in prevalence of hypertension in rural communities compared to that of the urban communities [5, 6]. This may be attributed to a growing increase in the age and lifestyle changes in the rural communities. Prevalence of hypertension in Nigeria has progressively increased from 10.1–13.3% and 8.9% in the late sixties to between 38.8 to 44.5% and 34.8% recently in rural and urban communities, respectively [2]. This difference in prevalence is partly due to changes in definition of hypertension from 160/95 mmHg earlier to 140/90 mmHg in JNC VII.

Most of these studies were done either in the South-West or the South-East Nigeria and very few were done in South-South, Nigeria. As a result of these identified changes in the epidemiologic trend of hypertension and its complications, there is therefore the need to regularly conduct a survey on the prevalence of hypertension.

This study was therefore conducted to find the recent prevalence of hypertension in both the rural and urban communities of Akwa Ibom state of Nigeria and also identify other risk factors associated with hypertension.

## 2. Subjects and Method

This is a cross sectional study of people in urban and rural communities for prevalence of hypertension and its predictors. Two senatorial zones were randomly selected from the 3 in the state. One local government was then randomly selected from the 9 or 10 local governments in each of the 2 selected senatorial zones. One urban community was selected in the local government and a rural community was identified. In the urban community, radio and TV advertorials were used to invite members of the community for a free screening program for hypertension and its risk factors. In the rural community, awareness for the program was created using town criers and church announcements.

The two rural communities were about 50 km apart and consist mainly of farmers while the distance between Uyo and Ikot Ekpene, the two urban centres, is 28.6 km. The distance between the Uyo urban and Use Abat was about 22 km while the distance between Ikot Ekpene urban and Asiak village was about 18 km.

Consented participants had their sociodemographic data, including age, sex, educational status, marital status, and occupation obtained. Their medical and family history of hypertension, diabetes, and renal disease were also obtained.

Subjects were fully examined and their anthropometricdata recorded. Weight and height were measured using a standard weighing scale and Seca stadiometer respectively. They had their Body mass index (BMI) calculated.

Abdominal and hip circumferences were measured using a flexible tape on a horizontal plane without compression of the skin. The blood pressure was taken using Aneroid sphygmomanometer on the right arm after 10-minute rest in a sitting position. The first and fifth phases of Korotkoff sounds were taken as systolic and diastolic blood pressure, respectively. Average two readings were recorded as the subject blood pressure. Blood pressure measurements were done by the same volunteer nurses and doctors in all the communities. Measurements were done between the hours of 9 am and 11 am.

Participants had urinalysis done with combur-10 test strips and random blood sugar test using Accu-Check Active glucometer manufactured by Roche India PVT Limited. Diabetes was defined based on a previous history of diabetes on drugs or random plasma glucose of ≥11.1 mmol/L. Hypertension was defined using the Seventh edition of Joint National Committee on detection, prevention, evaluation, and treatment of high blood pressure (JNCVII) of systolic BP ≥ 140 mmHg and diastolic BP ≥90 mmHg or patient is on drugs for control of hypertension. Prior knowledge of hypertension and diabetes was defined as participants that were diagnosed hypertensive and/or diabetic before the screening.

Ethical clearance was obtained from the University of Uyo Teaching Hospital ethical committee. Informed consent was obtained from all participants. Data analysis was performed using STATA 10, StataCorp, and College Station, Texas, USA. Multivariate logistic regression model was built using variables with *P* values of <0.25 at the univariate level or those known to have effect on blood pressure changes in a forward selection method. A receiver operator characteristic (ROC) curve was used to assess the utility of the final multivariate model. The final multivariate model was adjusted for the effect of age on the relationship between location of the participants and the risk of being hypertensive.

## 3. Results

A total of 1565 participants (590 urban, 978 rural) were recruited from the screening program over this period.  Table 1 details the sociodemographic and clinical characteristics of the participants while Table 2 compares the prevalence of cardiovascular disease risk factors in both groups. The independent risk factors for hypertension were age (6% increased risk for every one year increase), BMI (7% increased risk for every 1 kg/m^2^ increase), and proteinuria (59% increased risk for those proteinuric compared to the nonproteinuric).

## 4. Hypertension

**Figure 1:**
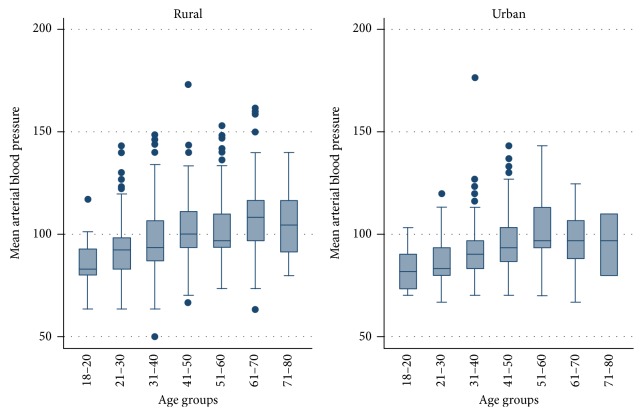
MABP distribution across age groups for both groups.

The prevalence of hypertension in the urban centers was lower than the rural centers (27.5% (95% CI 23.9–31.2%) versus 44.3% (95% CI 41.1–47.4%)). The awareness of personal hypertension status was low in both groups but significantly lower among the rural dwellers (Table 1). An awareness of a family history of hypertension was more prevalent among the urban dwellers than in the rural populace. Mean arterial blood pressure (MABP) was rising as age was increasing in both groups (Figure 1). On the average, the rural dwellers were older than the urban dwellers.

On multivariate logistic regression (Table 3), there was a higher risk of being hypertensive in the rural group than the urban group after adjusting for the effects of age, sex, body mass index, diabetic status, and proteinuria status.

## 5. Discussion

In this study we found a higher prevalence of hypertension in the rural communities than in the urban communities, 44.3% versus 27.5% (*P* < 0.001). This was not consistent with previous studies which demonstrated a higher prevalence of hypertension in urban societies than in rural societies [7, 8]. This may be attributed to a rise in westernized life style among the rural dwellers or it may be because the urban dwellers are more likely to be aware of their blood pressure status and, therefore, less likely to come out for the screening exercise. Also we found that rural dwellers were statistically older than the urban dwellers. This may have contributed significantly to the high prevalence of hypertension among them. This is because hypertension is known to increase with increasing age [8, 9]. There have been increasing trends in the prevalence of hypertension in the rural communities as demonstrated by recent studies ranging from 20.8% by Oladapo et al. [10], 23.6% by Andy et al. [11], to as much as 44.5% and 46.4% by Ahaneku et al. [12] and Onwubere et al. [5], respectively. These may be attributed to the increasing age of the rural communities as most young people prefer to migrate to the cities for white collar jobs while retirees moved in opposite direction back to the villages. This trend appears to be alarming and efforts should be made to check it before it is too late.

Our finding was slightly lower than that of Onwubere et al. [5]. This may be because they studied adult between the ages of 40–70 years while we included subjects from 18 years and above. However, our study had a higher prevalence of hypertension than that of Andy et al. [11] done two years earlier using almost the same population.

The difference between the urban and rural prevalence was maintained in this study even after predictors for hypertension such as age, obesity, sex, proteinuria, and diabetes were corrected using multivariate analysis (Table 3).

Genders were equally distributed in both urban and rural communities, although more women turned up for the screening exercise demonstrating the general poor attitude of men towards healthcare.

Urban dwellers were more likely to be obese as shown by higher BMI and waist circumference than the rural population. This may be because of sedentary life style and modernization of the urban societies.

There was no statistical significant risk of having hypertension between the male and female gender. This was not consistent with previous studies where men were found to be at a greater risk of being hypertensive [9, 13, 14]. This may be as a result of the relatively few numbers of males in this study. However a study by Adediran et al. [7] did not also find any statistically significant gender difference in tendency of being hypertensive in their study of hypertension prevalence in an urban and rural area of Nigeria.

Age and BMI were the most important predictors of hypertension in both rural and urban communities in this study. Increasing age has been found to be the most single predictor of hypertension [9, 14]. Also BMI had been found to be positively associated with hypertension in previous studies [7, 13]. The number of persons diagnosed with diabetes mellitus in the Urban communities was higher than the rural communities although this difference was not statistically significant. About 2.8% of the rural participants had diabetics mellitus which was lower than data from previous studies in Nigeria [15, 16].

## 6. Conclusion

These epidemiologic trends indicate that the hitherto gap that existed in prevalence of hypertension between the Urban-Rural communities is gradually being eroded and prevention strategies must be instituted urgently.

## Figures and Tables

**Table 1:** Sociodemographic and clinical characteristics of study participants.

	Urban (*N* = 590)	Rural (*N* = 978)	*P* value
Female gender (%)	73.7	70.1	0.12
Age (years)	39.9 ± 16.6	43.9 ± 11.9	<0.001
Educational level (%)			
No formal education	5.7	62.1	<0.001
Primary	4.7	26.3	
Secondary	16.1	8.8	
Tertiary	73.5	2.8	
Family history of hypertension (%)	24.1	4.6	<0.001
Family history of DM (%)	10.7	2.8	<0.001
BMI (kg/m^2^)	27.2 ± 5.8	22.5 ± 4.7	<0.001
Waist circumference (cm)	89.8 ± 13.3	78.9 ± 11.3	<0.001
Prior knowledge of HTN (%)	10.6	6.4	0.008
Prior knowledge of DM (%)	4.6	2.8	0.11

**Table 2:** Comparison of cardiovascular disease risk factors.

	Urban prevalence (95% CI)	Rural prevalence (95% CI)	*P*
Hypertension (%)	27.5 (23.9–31.2)	44.3 (41.1–47.4)	<0.001
Diabetes mellitus (%)	4.5 (2.8–6.3)	2.8 (1.6–4.1)	0.11
Obesity (%)	27.8 (24.1–31.4)	7.2 (5.4–8.9)	<0.001
Proteinuria (%)	21.8 (18.4–25.2)	26.2 (22.2–30.2)	0.09

**Table 3:** Univariate and Multivariate^*∗*^ regression models for hypertension predictors.

	Univariate Odds Ratio (95% CI) *P*-value	Multivariate Odds Ratio (95% CI) *P*-value
Location		
Urban	1	1
Rural	2.09 (1.67–2.62) <0.001	2.32 (1.66–3.24) <0.001
Age (years)	1.05 (1.04–1.06) <0.001	1.06 (1.05–1.08) <0.001
Sex		
Male	1	1
Female	0.99 (0.79–1.25) 0.96	1.03 (0.73–1.44) 0.88
BMI (kg/m^2^)	1.03 (1.01–1.05) 0.005	1.07 (1.04–1.10) <0.001
Diabetic status		
No	1	1
Yes	2.12 (1.15–3.89) 0.02	1.49 (0.70–3.19) 0.30
Proteinuric status		
No	1	1
Yes	1.47 (1.10–1.97) 0.01	1.59 (1.12–2.25) 0.009

^**∗**^Area under the ROC curve was 0.76.

## Limitations

This study was a one-spot screening study. Participants did not have opportunity for blood pressure recheck. There may also be the risk of selection bias as individuals in the urban centres who already knew their hypertensive or diabetic state may not have participated in the screening. More so in the rural areas, since the screening exercise was free, ill people may have been the ones to come out for screening therefore falsely inflating the prevalence of hypertension.
